# What do patients consider sensitive health information? A cross-sectional survey of national patient portal users

**DOI:** 10.1177/20552076261459512

**Published:** 2026-06-09

**Authors:** Saija Simola, Sari Kujala, Anna Kharko, Josefin Hagström, Charlotte Blease, Åsa Cajander, Rose-Mharie Åhlfeldt, Bo Wang, Bridget Kane, Maria Hägglund

**Affiliations:** 1 Department of Computer Science, 174277 Aalto University, Espoo, Finland; 2 Department of Women’s and Children’s Health, Participatory eHealth and Health Data Research Group, 8097 Uppsala University, Uppsala, Sweden; 3 Centre for Primary Care and Health Services Research, 5292 University of Manchester, Manchester, United Kingdom; 4 Department of Information Technology, 195561 Uppsala University, Uppsala, Sweden; 5 School of Informatics, 7359 University of Skövde, Skövde, Sweden; 6 Norwegian Centre for E-health Research, 60519 University Hospital of North Norway, Tromsø, Norway; 7 Business School, 4209 Karlstad University, Karlstad, Sweden; 8 59561 Uppsala University Hospital, Uppsala, Sweden

**Keywords:** patient portal, mental health, sensitive, medical information, notes, electronic health records, eHealth, national survey, patient accessible electronic health records

## Abstract

**Introduction:**

Patient-accessible electronic health records (PAEHRs) offer benefits, such as supporting self-management and care engagement. However, some patients, particularly those with mental health conditions, might experience negative emotions such as worry when reading unexpected or sensitive information in their PAEHRs.

**Methods:**

A web-based survey of 4459 respondents distributed via the Finnish national patient portal included multiple-choice and open-ended questions. Respondents consisted of two patient groups who had received care either for 1) mental health or 2) other conditions. Inductive content analysis was performed to explore the kind of information that was perceived as sensitive in the PAEHR. Associations between sociodemographic factors including the type of care and reporting health information as sensitive were calculated via the multivariable binary logistic regression analysis.

**Results:**

Mental health (61.3%), and intimate health (8.3%) were the most frequently mentioned as especially sensitive types of information among respondents, who also stressed that the sensitive nature of the health information depended on the context. Within the mental health information type, therapy or treatment was most often mentioned (3.5%) as sensitive. Respondents who had received mental health care were significantly more likely to perceive certain information as sensitive (53.2%) than other patients (28.8%; Adjusted OR=2.783, 95% CI=[2.333, 3.319], p<0.001).

**Conclusions:**

This study delves into the sensitive character of mental health information within PAEHR. The sensitivity of information also depends on the consequences for the patients when data will be used in another context. Documenting sensitive information carefully and safeguarding it is recommended to maintain trust in electronic health records and healthcare.

## Introduction

In recent decades, many countries, including the Nordic countries, have offered patient-accessible electronic health records (PAEHRs) via national patient portals.^1^ Studies have shown that patients’ access to their electronic health records (EHRs) offers many benefits to patients, family caregivers, and health care professionals (HCPs). PAEHRs increase communication between patients and HCPs^2,3^ and help patients better remember^4–7^ and understand^8,9^ their care process. Furthermore, PAEHRs increase patients’ satisfaction,^4^ trust,^10^ participation,^5,8^ and engagement^6,9^ with care. With shared access to EHRs, family caregivers can support their vulnerable family members within health care.^3,11^ PAEHR helps family caregivers coordinate care despite geographical distance and feel less stressed.^11^ Nevertheless, patients’ access to EHRs varies internationally,^12^ and access can be limited depending on the type of data,^13^ such as psychiatry notes. In general, patients desire access to their records^14^ even though notes might raise some challenges, such as feeling offended,^9^ and mental health notes might increase worry, stress or feelings of upset.^15,16^

Furthermore, patients with mental health conditions^17,18^ and patients with serious or potentially stigmatizing conditions such as depression or sexual infections^19^ report concerns related to privacy, security, and unwanted access to their EHRs. Shen et al.^20^ found that patients’ previous experiences of privacy affect trust in mental health contexts. Acquisti et al.^21^ stated that attitudes toward privacy are subjective and depend on the context. Consequently, patients might have different experiences about sensitive information and privacy.^21^

Furthermore, adolescents might be concerned about sensitive information documented in the EHR, such as sexually transmitted diseases, prevention, or mental health, preventing them from seeking care or discussing these topics.^22^ Moreover, sharing data through proxy access raises ethical concerns, particularly for sensitive information in connection with mental health, between parents and their children or adolescents.^10,22^ In contrast, similar concerns arise when adult children or partners take care of adults with mental health conditions and may be able to access to other persons’ health information in EHR with proxy-access.^10^

HCPs have shared patients’ concerns, particularly those related to psychiatric patients and records.^4,23^ Professionals are even more worried than patients about the sensitivity of certain notes.^24^ Psychiatric HCPs are concerned about patients’ potential negative emotions and reactions after reading their PAEHR, such as worry, offense, or disagreement.^4,25–27^ Concerns about the security of sensitive data may lead HCPs to omit necessary but sensitive details from records^28,29^ or to avoid directly reporting potentially stigmatizing laboratory results.^30^ Some studies report that HCPs still document information they deem necessary for continuity of care at the risk of offending a patient.^10,14,31^ However, another study reported that HCPs might be reluctant to share certain sensitive topics or parts of the notes with patients at all.^14^ Professionals have reported that they have generalized^32^ or changed their notes,^33^ especially regarding sensitive topics^34,35^ such as mental health, cancer, or substance abuse.^34,36^ Thus, they might be using more time to write their notes.^27,35,37^

Nissenbaum^38^ has defined sensitive information in general as “information whose collection, disclosure, or use may result in harm to its subjects”. Additionally, they stated that highly sensitive information needs privacy protection.^38^ In some previous studies, sensitive information has been defined broadly in mental health contexts,^39,40^ and in some cases, all mental health information has been conceived as sensitive.^40^ Although, there are multiple definitions of sensitive information, it is common for legislators and policy makers to consider all health information sensitive (e.g.^41^). Thorarensen^42^ and Mantas et al.^43^ stated that health information is usually personal data, which are commonly considered sensitive information. By the same token, definitions of sensitive health data may vary depending on legislation and policies.^42,43^

Soni et al.^44^ reported that patients’ perspectives vary, and individual perceptions of sensitive health information should be explored. The aim of this study is to explore patients’ perspectives and identify which types of information in PAEHRs are perceived as especially sensitive without predefined options. The hypothesis is that patients perceive mental health information as sensitive. Thus, this study aims to explore what kind of mental health information is perceived as sensitive.

Leveille et al.^45^ reported that diverse patient groups, including vulnerable patient groups, should be better understood to improve PAEHR design. To support the adoption and development of PAEHRs, strengthen patients’ trust, and improve HCPs’ work, it is essential to understand how patients perceive the sensitivity of health information and whether certain groups have specific needs. Since patients and HCPs have described sensitive nature of mental health information, the hypothesis is that patients who have received mental health care are more likely to experience certain information as sensitive. This study examines whether some patient groups are more likely to experience health information as sensitive. The focus of this study is on patients who have received mental health care or other health care. In addition, the sociodemographic factors are investigated.

Research questions:

RQ1. What do patients perceive as sensitive health information in their PAEHR?
RQ2. What mental health related information patients perceive as sensitive in their PAEHR?
RQ3. How are the patients’ mental or other health care and sociodemographic factors associated with the reporting particular health information as sensitive in their PAEHR?

This study explores patients’ experiences with sensitive health information. To examine whether some types of information are perceived as *especially sensitive*, we allowed patients to define sensitivity themselves rather than selecting from predefined options. This approach avoids leading respondents and captures their own perspectives.

## Methodology

### Aim, design and setting of the study

This study explores sensitive information in the PAEHR with a focus on patients who have received mental health or other health care and can access this information through the national patient portal, My Kanta, in Finland. All Finnish citizens have access to PAEHR via the national patient portal. HCPs have access to the same health information via their own professional portals.

The responses were collected via a web-based survey of logged-in patient users between January 24, 2022, and February 14, 2022, in both official languages in Finland, Finnish and Swedish. The survey was part of the NORDeHEALTH 2022 Patient Survey, and the overall survey data collection, including the rationale for collecting demographic data, is described in more detail in Hägglund et al.^1,46^ The survey covered multiple research questions, and questions on the respondents’ demographics therefore needed to be rather extensive.

The survey followed the CHERRIES (Checklist for Reporting Results of Internet E-Surveys; See Supplementary file “CHERRIES”).^47^ When logging out from the PAEHR, the respondents were invited to voluntarily fill out the anonymous questionnaire, which consisted of multiple-choice and open-ended questions, hosted online by Webropol (version 3.0). None of the questions were mandatory to answer. This study focused on patients’ perspectives. Some of the respondents had health care professional education. Those participants were asked to respond to this survey as a patient, even though they would have health care professional education as well. The participants’ survey responses were not in any way linked to their PAEHRs in the national patient portal.

A mixed-methods approach was employed in this research. The survey was chosen because it provides a large sample size to explore the differences between patient groups. Moreover, the collection of qualitative responses allowed us to gain a deeper understanding of sensitive information. To explore whether patients find certain health information to be especially sensitive, the respondents were asked *“Do you consider some types of health information especially sensitive?”*. If the respondent selected “*yes*”, the following open-ended question was asked to identify what constitutes this especially sensitive information: *“Can you give an example of what type of health information is most sensitive to you?”.* The predefined options were used for collecting background information only to avoid leading the respondents when asking what kind of information they described as especially sensitive. The respondents were asked a variety of questions about their sociodemographic factors, including age, gender, overall health, education, having received care in the past 24 months, and having an HCP education (See Supplementary file “Survey Questions”). The survey was anonymous and provided an easier way to describe challenging topics related to especially sensitive health information in their own language.

**Figure 1.**
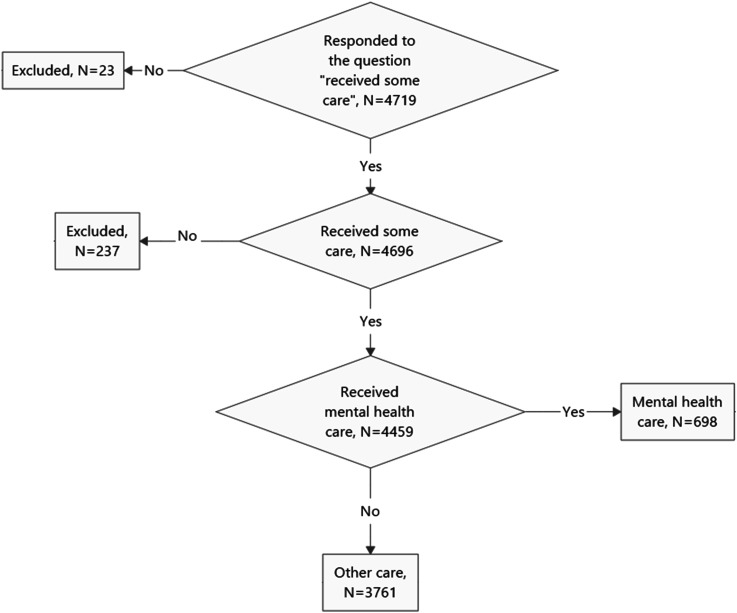
Type of care exclusion and inclusion criteria.

Figure 1 illustrates the groups’ inclusion and exclusion criteria. The groups were 1) respondents who reported having received mental health care in the past two years and 2) respondents who reported any other type of care in the past two years. The respondents who reported having received both mental health and any other type of care were included in the mental health care group. Additionally, respondents who did not respond to the question about the type of care received or had not received any care in the past two years were excluded (N=260). The initial sample consisted of 4719 respondents, 4459 of whom had received mental health or other care.

### Characteristics of the respondents

The respondents were more often female (73.1%; Table 1). More than one-fifth of the respondents had HCP education themselves (22.0%). The larger group consisted of patients who had received other care, representing 84.3% of the respondents. The other group included patients having received mental health care, accounting for 15.7% of the respondents. The respondents who had received other care were relatively older; 78.8% of them were 55 years or older, whereas 40.7% of those who had received mental health care were 55 years or older.

**Table 1.** Characteristics of the respondents based on type of received care.

N (%)		Mental health, N (%)	Other conditions, N (%)	Total, N (%)
		698 (15.7)	3761 (84.3)	4459 (100)
Health condition	Mean, 1 = Very good, 5 = Very bad	3.15 (96.7)	2.74 (98.9)	2.81 (98.6)
	I don’t know/I don’t want to answer	20 (2.9)	35 (0.9)	55 (1.2)
	No response	3 (0.4)	7 (0.2)	10 (0.2)
Age	15 – 17 years	10 (1.4)	6 (0.2)	16 (0.4)
	18 – 19 years	3 (0.4)	5 (0.1)	8 (0.2)
	20 – 24 years	34 (4.9)	23 (0.6)	57 (1.3)
	25 – 34 years	96 (13.8)	111 (3.0)	207 (4.6)
	35 – 44 years	122 (17.5)	215 (5.7)	337 (7.6)
	45 – 54 years	141 (20.2)	422 (11.2)	563 (12.6)
	55 – 64 years	185 (26.5)	924 (24.6)	1109 (24.9)
	65 – 74 years	73 (10.5)	1427 (37.9)	1500 (33.6)
	75 – 84 years	25 (3.6)	566 (15.0)	591 (13.3)
	85 years or more	1 (0.1)	46 (1.2)	47 (1.1)
	No response	8 (1.1)	16 (0.4)	24 (0.5)
Gender	Female	555 (79.5)	2705 (71.9)	3260 (73.1)
	Male	121 (17.3)	1016 (27.0)	1137 (25.5)
	Other/I don’t want to answer	20 (2.9)	33 (0.9)	53 (1.2)
	No response	2 (0.3)	7 (0.2)	9 (0.2)
Education	No formal education,	3 (0.4)	13 (0.3)	16 (0.4)
	Elementary school,	59 (8.5)	389 (10.3)	448 (10.0)
	12 years school - Upper secondary education,	224 (32.1)	930 (24.7)	1154 (25.9)
	Higher vocational education (vocational diploma),	111 (15.9)	877 (23.3)	988 (22.2)
	Higher education ≤ 3 years (first cycle -bachelor),	149 (21.3)	641 (17)	790 (17.7)
	Higher education, >3 years (second cycle-master),	120 (17.2)	708 (18.8)	828 (18.6)
	Research, (third cycle) of higher education	7 (1.0)	69 (1.8)	76 (1.7)
	Something else	17 (2.4)	77 (2.0)	94 (2.1)
	No response	8 (1.1)	57 (1.5)	65 (1.5)
Has health care professional education	Yes	168 (24.1)	813 (21.6)	981 (22.0)
	No	513 (73.5)	2866 (76.2)	3379 (75.8)
	No response	17 (2.4)	82 (2.2)	99 (2.2)
Employment	Full time	165 (23.6)	897 (23.9)	1062 (23.8)
	Part time	75 (10.7)	185 (4.9)	260 (5.8)
	Student	64 (9.2)	68 (1.8)	132 (3.0)
	Retired	201 (28.8)	2285 (60.8)	2486 (55.8)
	Unemployed	74 (10.6)	131 (3.5)	205 (4.6)
	Not able to work	80 (11.5)	75 (2.0)	155 (3.5)
	None of the above (free text)	36 (5.2)	104 (2.8)	140 (3.1)
	No response	3 (0.4)	16 (0.4)	19 (0.4)

### Analysis process

#### Qualitative inductive content analysis

**Figure 2.**
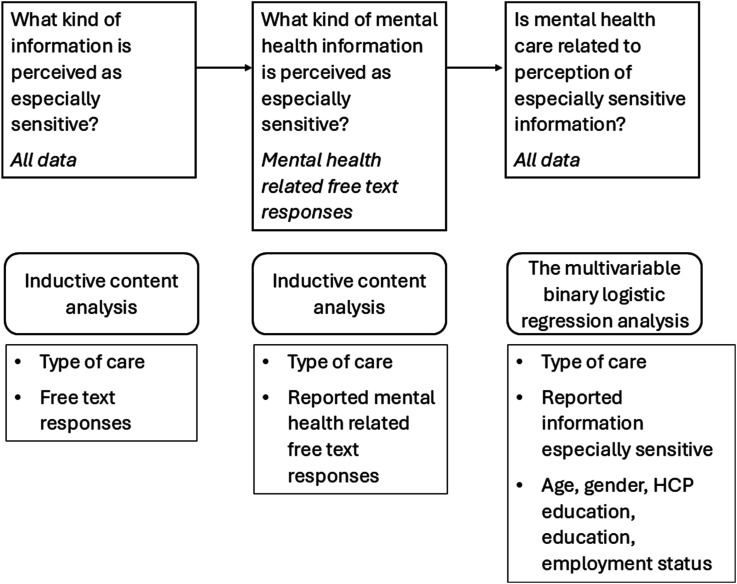
The analysis process.

Figure 2 presents an overview of the analysis process. To explore RQ1 and RQ2, the study used qualitative analysis to gain insights into patients’ experiences of especially sensitive health information. STROBE Statement Checklist^48^ provide as appendix (See Supplementary file “STROBE”). Qualitative inductive content analysis was conducted via the Atlas. TI (version 25.0.1.32924) following the framework by Elo & Kyngäs.^49^ If responses included identification information, it was removed from the data to protect participants’ identities. All data collected was analyzed.

The inductive content analysis was chosen to explore sensitive information based on patients’ responses without predefined themes or options. The first author read through the data and performed the iterative qualitative analysis. All open-ended responses were coded via in vivo coding. The second author verified the codes. Preliminary codes with similar meanings were merged into a smaller set of codes. Final codes were grouped into larger themes. Both authors have prior experience of qualitative analysis. Each response had one or more different codes. The number of respondents included in each code was calculated. Authors coded all unclear responses and created larger themes in collaboration. If authors disagreed about coding decisions this was discussed until agreement was obtained.

To further deepen our understanding of what mental health information was considered especially sensitive, a second round of content analysis was conducted, with a more in-depth examination of the responses related to mental health information identified in the first-round analysis. The category “not specified” was used to indicate that the response did not include details regarding mental health information but rather mentioned “mental health” or “mental health information” in general as especially sensitive. If the response contained information not related to mental health, it was not included in the second-round analysis.

#### Statistics

Data management and statistical analysis were performed via Excel (Version 2502) and SPSS (Version 30.0.0.0). To address RQ3, patients were categorized into groups on the basis of whether they had received mental health or other care (Figure 1). The observations were independent. The dependent binary variable was whether patients have reported sensitive information. Independent categorial variables were type of received care (past 2 years), age, gender, has HCP education, education, and employment status.

Analysis was presented with multivariable binary logistic regression analysis, since the dependent variable “having reported sensitive notes” was binary, and independent background variables were categorical.^50,51^ The logistic regression analysis was calculated in two steps. Firstly, Model A included variable type of received care based on hypothesis. Secondly, Model B included Model A’s variable, and age, gender, has HCP education, education, and employment status. Model A and B included a single dummy variable if the respondent received any mental health care in the past two years. Since all respondents had received some care, false on this indicated they received other care.

The null hypothesis was that having reported sensitive notes does not significantly depend on the type of care they have received. A p value <0.05 was considered significant. The multicollinearity was tested calculating variance inflation factors (VIF) for the independent variables. VIF values ranged from 1.000 to 1.448, which indicates that multicollinearity was not a concern in this study.^52–54^

## Results

### Health information considered especially sensitive

Most patients (1296/1446, 89.6%; Table 2) who considered some information especially sensitive specified the type of health information. The themes are summarized in Table 2, with mental health information emerging as the most frequently mentioned information regardless of the type of care received. Additionally, nearly one in five patients (227/1296, 17.5%; Table 2) described all health information as sensitive.

**Table 2.** The type of health information reported as especially sensitive.

n (%)	334 (25.8)	962 (74.2)	1296 (100)
	Mental health care, n (%)	Other care, n (%)	Total, n (%)
**Information relating conditions**			
Mental health	251 (75.1)	543 (56.4)	794 (61.3)
Serious conditions	5 (1.5)	29 (3.0)	34 (2.6)
Cancer and tumors	0 (0)	14 (1.5)	14 (1.1)
Diagnosis (MH excl.)	1 (0.3)	4 (0.4)	5 (0.4)
**Type of information**			
All health information	40 (12.0)	187 (19.4)	227 (17.5)
Old or unnecessary information	7 (2.1)	22 (2.3)	29 (2.2)
Wrong information	1 (0.3)	11 (1.1)	12 (0.9)
Information from social services	0 (0)	3 (0.3)	3 (0.2)
**Sexuality and reproductive medicine**			
Intimate health	26 (7.8)	81 (8.4)	107 (8.3)
Sexuality	23 (6.9)	50 (5.2)	73 (5.6)
Sexually transmitted diseases	19 (5.7)	51 (5.3)	70 (5.4)
Abortion	4 (1.2)	13 (1.4)	17 (1.3)
Childlessness and infertility treatment	6 (1.8)	10 (1.0)	16 (1.2)
LGBTQIA+	1 (0.3)	11 (1.1)	12 (0.9)
Heredity and genetics	2 (0.6)	8 (0.8)	10 (0.8)
Maternal health	1 (0.3)	6 (0.6)	7 (0.5)
**Information with possible consequences**			
Sensitive depending on who has access to the information	9 (2.7)	49 (5.1)	58 (4.5)
Stigmatic conditions	15 (4.5)	28 (2.9)	43 (3.3)
Harmful in future/another context	12 (3.6)	21 (2.2)	33 (2.5)
Patient’s disagreement of care	0 (0)	2 (0.2)	2 (0.2)
**Information relating specific theme**			
Use of alcohol and drugs	8 (2.4)	27 (2.8)	35 (2.7)
Medications and prescriptions	4 (1.2)	18 (1.9)	22 (1.7)
Family and relationships	6 (1.8)	14 (1.5)	20 (1.5)
Crimes	4 (1.2)	7 (0.7)	11 (0.8)
Weight	6 (1.8)	3 (0.3)	9 (0.7)
Memory disorder	0 (0)	6 (0.6)	6 (0.5)
HCP’s description of appearance	2 (0.6)	4 (0.4)	6 (0.5)
Vaccination	0 (0)	3 (0.3)	3 (0.2)
Living will	0 (0)	2 (0.2)	2 (0.2)

The results present the frequency of mentions for specific information codes within the especially sensitive free-text answers. Codes with only 1 response in total were excluded.

#### Types of sensitive health information

Information related to sexuality, including intimate health, sexually transmitted diseases, and LGBTQIA+, was frequently mentioned as especially sensitive health information (Table 2). The respondents offered sensitive details, such as *“Erectile dysfunction.” (Respondent #0051, other care)* and *“Sexual orientation and gender expression (e.g., trans background and intersex).” (Respondent #3949, mental health care)*. One common theme was reproductive health and care, including various situations: *“e.g. a young person’s unexpected pregnancy and a possible abortion.” (Respondent #0094, other care).*

In addition, patients reported other types of information that were considered especially sensitive, such as information relating to other people, e.g. *“Information about family/relatives, i.e., information about others” (Respondent #0866, other care),* and *“Partner abuse, i.e., sexual and physical violence.” (Respondent #3915, mental health care).*

Patients described how old, or unnecessary information is sensitive: *“All that are unrelated to the current issue. For example, if I break my wrist, the health care staff does not need to read the information about the gynecologist visit.” (Respondent #0324, other care).* In addition, wrong information can also be considered sensitive: *“The kind of information that has already been proven numerous times as an incorrect diagnosis, medical malpractice and wrong medication, but which health professionals refuse to remove from my information, even if they could.” (Respondent #0833, other care).*

#### Context of sensitive health information

The sensitivity of certain information seemed to depend on who would have access to it: *“Sharing with occupational health may not always be appropriate.” (Respondent #0155, other care),* or how it will be used: *“Personal information in the wrong hands. Can lead to crimes, e.g., extortion.” (Respondent #0122, other care)*.

Concerns were expressed about conditions that might be stigmatized, such as “*degenerative diseases (e.g., MS). That is, diseases that can change the way others treat me, either at work or within friends and family.” (Respondent #3659, mental health care)*, and how those might increase the negative emotions “*Health information that is related to its own kind of shame and stigmatization*” (*Respondent #3964, mental health care).* Additionally, respondents described how certain health information might have broader impact in their life, when other people will perceive and treat them differently: *“For example, my memory disorder, because it may affect the treatment I receive and cause prejudice in other people.” (Respondent #0081, other care).*

Respondents were worried about how certain information may impact even patients’ employment opportunities or insurance and added experiences of discrimination *“Related to serious illnesses, which exposes you to discrimination in working life or can affect, e.g., the decisions of a bank or an insurance company.” (Respondent #4424, other care)*. Patients also appreciated sensitive handling of their information throughout the care process: *“Rape, for which I am currently getting mental health treatment. (HCPs) do not know how to handle it sensitively.” (Respondent #3943, mental health care).*

### Types of especially sensitive mental health information

As shown in Table 2, more than half of the patients (56.4%-75.1%) described mental health information as especially sensitive, despite the type of care they had received. To enhance the understanding of whether certain mental health information is especially sensitive, researchers continued analyzing mental health information.

Further analysis (794/1296, 61.3%; Tables 2 and 3) revealed that most respondents described all mental health information as especially sensitive or that mental health-related information was generally mentioned as especially sensitive (Table 3). Therapy or treatment and mental disorders or diagnoses were the most frequently mentioned specific sensitive mental health information.

**Table 3.** The type of mental health information reported as especially sensitive.

n (%)	251 (31.6)	543 (68.4)	794
	Mental health care, n (%)	Other care, n (%)	Total, n (%)
**Not specified**	184 (73.3)	430 (79.2)	614 (77.3)
**Specific information**			
Therapy or treatment	15 (6.0)	13 (2.4)	28 (3.5)
Mental disorder or diagnosis	9 (3.6)	18 (3.3)	27 (3.4)
Content of the visit	10 (4.0)	7 (1.3)	17 (2.1)
Depression	8 (3.2)	6 (1.1)	14 (1.8)
Visit itself	4 (1.6)	9 (1.7)	13 (1.6)
Ability to work	0 (0)	9 (1.7)	9 (1.1)
Psychiatric evaluation	4 (1.6)	4 (0.7)	8 (1.0)
Serious condition	1 (0.4)	4 (0.7)	5 (0.6)
HCP’s interpretation	2 (0.8)	2 (0.4)	4 (0.5)
Mental health medication	1 (0.4)	3 (0.6)	4 (0.5)
Trauma	1 (0.4)	3 (0.6)	4 (0.5)
Addiction	0 (0)	3 (0.6)	3 (0.4)
Mental health issues related to another disease	2 (0.8)	1 (0.2)	3 (0.4)
Eating, panic or personality disorder	2 (0.8)	1 (0.2)	3 (0.4)
Insomnia	0 (0)	2 (0.4)	2 (0.3)
**Worry on potential consequences**			
Stigmatizing	9 (3.6)	13 (2.4)	22 (2.8)
Affects other care	10 (4.0)	11 (2.0)	21 (2.6)
Visible after recovery	4 (1.6)	5 (0.9)	9 (1.1)
Affects working life	2 (0.8)	5 (0.9)	7 (0.9)
If visible for somatic care	2 (0.8)	2 (0.4)	4 (0.5)
Affects possibility to get insurance	2 (0.8)	2 (0.4)	4 (0.5)
If visible for authorities	0 (0)	2 (0.4)	2 (0.3)
Temporary mental health care	0 (0)	2 (0.4)	2 (0.3)

Codes with only 1 response in total were excluded.

The respondents also described mental health information as especially sensitive due to potential consequences. Patients were worried about the stigmatizing nature of mental health related information or how it might affect their care when seeking help for somatic conditions. For example, one respondent who had received mental health care describes *“Depression still marks a person in the eyes of many doctors, and the person’s other problems are blamed on it” (Respondent #3718, mental health care)*. In addition, another respondent who had received other care reported: *“A person’s mental health information because it easily stigmatizes, and other people behave attitudinally” (Respondent #128, other care).*

### Associations between type of care and especially sensitive information

As shown in Table 5, patients who had received mental health care reported statistically significantly more likely that some information was especially sensitive than patients who received other types of care (Adjusted OR = 2.783, 95% CI = [2.333, 3.319], p <0.001; Model A; Table 5). Over half of the patients who had received mental health care reported that some health information was especially sensitive (368/692, 53.2%; Table 4 and see Supplementary file “Background Information Sensitive”), whereas with less than one-third of the other respondents shared that experience (1078/3738, 28.8%; Table 4 and see Supplementary file “Background Information Sensitive”). This statistically significant association with received care persisted even after including additional variables in Model B.

**Table 4.** Characteristics of respondents who reported some information as sensitive.

Sensitive		Total, n (%)	
		Yes	No
Age	15 – 17 years, N (%)	7 (43.8)	9 (56.3)
	18 – 19 years, N (%)	3 (37.5)	5 (62.5)
	20 – 24 years, N (%)	39 (68.4)	18 (31.6)
	25 – 34 years, N (%)	101 (48.8)	106 (51.2)
	35 – 44 years, N (%)	151 (45.2)	183 (54.8)
	45 – 54 years, N (%)	261 (46.5)	300 (53.5)
	55 – 64 years, N (%)	399 (36.3)	699 (63.7)
	65 – 74 years, N (%)	372 (25.0)	1117 (75.0)
	75 – 84 years, N (%)	96 (16.3)	493 (83.7)
	85 years or more, N (%)	8 (17.0)	39 (83.0)
	No response, N (%)	9 (37.5)	15 (62.5)
Gender	Female	1107 (34.2)	2131 (65.8)
	Male	295 (26.1)	836 (73.9)
	Other or no response, N (%)	44 (72.1)	17 (27.9)
Has health care professional education	Yes	368 (37.7)	608 (62.3)
	No	1045 (31.1)	2311 (68.9)
	No response, N (%)	33 (33.7)	65 (66.3)
Education	No formal education	3 (20.0)	12 (80.0)
	Elementary school	77 (17.3)	368 (82.7)
	12 years school - Upper secondary education	352 (30.8)	792 (69.2)
	Higher vocational education (vocational diploma)	275 (28.1)	703 (71.9)
	Higher education ≤ 3 years (first cycle -bachelor)	312 (39.6)	476 (60.4)
	Higher education, >3 years (second cycle-master)	350 (42.3)	477 (57.7)
	Research, (third cycle) of higher education	32 (42.7)	43 (57.3)
	Something else	21 (22.3)	73 (77.7)
	No response	24 (37.5)	40 (62.5)
Employment status	Full time	420 (39.7)	639 (60.3)
	Part time	102 (39.8)	154 (60.2)
	Student	76 (57.6)	56 (42.4)
	Retired	610 (24.7)	1859 (75.3)
	Unemployed	87 (42.9)	116 (57.1)
	Not able to work	75 (48.4)	80 (51.6)
	Something else	68 (49.6)	69 (50.4)
	No response	8 (42.1)	11 (57.9)
Received care	Mental health care	368 (53.2)	324 (46.8)
	Other care	1078 (28.8)	2660 (71.2)
Total	​	1446 (32.6)	2984 (67.4)

Female patients (Adjusted OR = 1.209, 95% CI = [1.018, 1.435], p = 0.030; Model B; Table 5) were significantly more likely to report sensitive information. Additionally, older patients (75 – 84 years old; Adjusted OR = 0.223, 95% CI = [0.067, 0.746], p = 0.015; Model B; Table 5 and Supplementary file “Background Information Sensitive”) were significantly less likely to report that some information was especially sensitive.

**Table 5.** Associations between having reported especially sensitive information and sociodemographic factors.

Variables		Model A			Model B		
		Coefficient B (SE)	p-value	Adjusted OR (95% CI)	Coefficient B (SE)	p-value	Adjusted OR (95% CI)
Received mental health care (Ref.cat: No)	Yes	**1.023 (0.090)**	**<0.001**	**2.783 (2.333, 3.319)**	**0.654 (0.101)**	**<0.001**	**1.923 (1.577, 2.345)**
Age (Ref.cat: 15 – 17 years)	18 – 19 years	​	​	​	-0.470 (0.913)	0.607	0.625 (0.104, 3.743)
	20 – 24 years	​	​	​	0.615 (0.638)	0.335	1.850 (0.530, 6.463)
	25 – 34 years	​	​	​	-0.324 (0.591)	0.583	0.723 (0.227, 2.304)
	35 – 44 years	​	​	​	-0.350 (0.597)	0.558	0.705 (0.218, 2.273)
	45 – 54 years	​	​	​	-0.123 (0.597)	0.837	0.885 (0.274, 2.851)
	55 – 64 years	​	​	​	-0.472 (0.596)	0.428	0.624 (0.194, 2.004)
	65 – 74 years	​	​	​	-0.951 (0.607)	0.117	0.386 (0.117, 1.270)
	75 – 84 years	​	​	​	**-1.500 (0.616)**	**0.015**	**0.223 (0.067, 0.746)**
	85 years or more	​	​	​	-1.088 (0.735)	0.139	0.337 (0.080, 1.422)
Gender (Ref.cat: Male)	Female	​	​	​	**0.190 (0.088)**	**0.030**	**1.209 (1.018, 1.435)**
Has health care professional education (Ref.cat: No)	Yes	​	​	​	0.077 (0.086)	0.375	1.080 (0.912, 1.279)
Education (Ref.cat: No formal education)	Elementary school	​	​	​	-0.090 (0.699)	0.897	0.914 (0.232, 3.592)
	12 years school - Upper secondary education	​	​	​	0.475 (0.691)	0.491	1.609 (0.415, 6.232)
	Higher vocational education (vocational diploma)	​	​	​	0.585 (0.692)	0.398	1.795 (0.462, 6.967)
	Higher education ≤ 3 years (first cycle -bachelor)	​	​	​	0.844 (0.693)	0.223	2.326 (0.598, 9.044)
	Higher education, >3 years (second cycle-master)	​	​	​	1.123 (0.692)	0.105	3.075 (0.792, 11.943)
	Research, (third cycle) of higher education	​	​	​	1.370 (0.734)	0.062	3.934 (0.933, 16.589)
Employment status (Ref.cat: Not able to work)	Full-time	​	​	​	-0.357 (0.190)	0.061	0.700 (0.482, 1.016)
	Part-time	​	​	​	-0.350 (0.222)	0.115	0.704 (0.456, 1.089)
	Student	​	​	​	0.127 (0.287)	0.658	1.135 (0.647, 1.993)
	Retired	​	​	​	-0.208 (0.207)	0.314	0.812 (0.541, 1.218)
	Unemployed	​	​	​	-0.100 (0.232)	0.668	0.905 (0.575, 1.426)
Constant	​	**-0.940 (0.038)**	**<0.001**	**0.390**	-0.812 (0.891)	0.362	0.444

Twenty-nine respondents (0.7%) did not respond, including 6 (0.9%) representing those who had received mental health care and 23 (0.6%) representing those who had received other care. Significant p values (p <0.05) are in bold.

There was no significant difference in education, employment status, or whether the patient has an HCP education themselves. The model’s fit was evaluated using Cox and Snell’s R^2^ (Table 6), which was calculated to be 0.031 (Model A) and 0.089 (Model B). This suggests that extended Model B accounts for more variance, but explanatory power for the variability in the dependent variable is modest in both models.

**Table 6.** Evaluations of models’ fit.

Model	χ^2^	df	p-value	χ^2^ (Hosmer-Lemeshow)	df (Hosmer-Lemeshow)	p-value (Hosmwe-Lemeshow)	-2 Log Likelihood	Cox & Snell R^2^	Nagelkerke R^2^	Variables
Model A	128.022	1	<0.001	0	0	-	4881.671	0.031	0.044	Received mental health care
Model B	247.034	22	<0.001	4.244	8	0.835	4634.637	0.089	0.125	Age, Received mental health care, Gender, Has health care education, Education, Employment status

## Discussion

This study explored the types of health information in PAEHR that might be sensitive. Mental health, intimate health, sexuality, and sexually transmitted diseases were the most reported. Respondents also described that certain information may be sensitive if it could be harmful in another context. Therapy or treatments, mental disorders or diagnoses, and the content of the visit were the most common sensitive mental health information. In our study sample, more than half of the patients who had received mental health care considered certain information to be especially sensitive. In contrast, more than one-fourth (28.8%) of the patients who had received other care shared that expression.

Mental health information was most frequently identified as especially sensitive across respondents in all patient groups. This finding is consistent with previous research.^40,44,55^ Previous studies have identified similar sensitive topics that our results show, such as sexuality,^44,56^ reproductive health,^56^ and intoxicants or drugs.^44,56–58^ Particularly among adolescents, mental health and sexually transmitted diseases have been reported to be sensitive.^59,60^

In accordance with Nissenbaum’s^38^ idea that use of information may affect the experience of sensitivity, this study explored that besides the types of health information the context of the health information may be meaningful for the respondents. In other words, respondents also described concerns about how health information may be harmful in certain situations, such as unwanted access or misuse, not only the information itself.

Some respondents spontaneously explained that information sensitivity depends on who can access it or its potential harmfulness in the future. Thus, this study may offer implications for future research for exploring negative consequences of sensitive health information. When developing EHRs, those potential negative impacts of sensitive information could be decreased.

The open-ended responses of this study provide preliminary insight into why mental health information may be perceived as sensitive and how patients might experience it, complementing earlier findings.^39^ Worry about how mental health information might be labeling and affect other care or working life was also often reported by patients. The potentially stigmatizing nature of mental health information were reported also in previous research.^58,61^ Health care professionals have shared our respondents’ concern about the stigmatizing nature of mental health, which may potentially affect other care.^62^ Additionally, our results reflect those of Marwaha & Johnson’s^63^ findings that people hesitate to share mental health information with employers due to potential stigma. Concerns about potential harm due to information misuse in other contexts, such as job seeking or unwanted access, have also been described in earlier studies.^64^

The experience of whether some health information is sensitive decreased with age among respondents. This may reflect younger patients’ greater awareness of digital privacy or concerns about how such information could affect future employment or education opportunities. Additionally, female respondents were more likely to experience information as sensitive.

Three-quarters of the respondents who had received mental health care (75.1%) described mental health information as especially sensitive, compared to over half of those who had received other care (56.4%). Previous studies have shown that patients with mental health conditions are experiencing shame and might be less likely to receive help or medical treatment.^65^ Despite potential consequences, patients who had received mental health care reported that access to their PAEHRs supports trust in HCPs and communication with them.^66^

Generally, it may be unclear who owns the patient’s health information and, ultimately, who is responsible for deciding how certain sensitive information could be managed. The previous studies highlight shortcomings in information management from privacy perspectives.^67^ Additionally, privacy attitudes are subjective and content and context dependent.^21^ However, based on GDPR^41^ patients have right to access their health information which should be confidentially managed and accurate.

Furthermore, HCPs have shared patients’ experiences that certain topics may be sensitive, such as mental health and illness, intoxicants,^24,34,57,68^ LGBTQIA+, sexuality, diagnostic reasoning,^68^ abortion,^69^ and body size.^24,34^ HCPs have expressed concerns and discomfort about inquiries related to gender identity.^70,71^ However, Soni et al.^57^ reported that the sensitivity level of mental health information varies between HCPs and patients. Moreover, the HCPs perspective of sensitive information could be more explored.

The study also provides valuable insight into what sensitive information may be pertinent to patients who may not want to share certain parts of their EHR with their guardians or caregivers. With proxy access, informal caregivers or family members have access to records.^13^ These findings suggest that some of the sensitive information might be sensitive depending on who has access to it or if it relates to other people, such as maternal health, heredity, genetics, crimes, or information concerning family and relationships. Such circumstances could be handled carefully when sensitive health information is shared with caregivers, who might use the information in an unwanted way or have potentially negative emotions that might affect caregivers’ and patients’ relationships. As EHRs might be available via proxy access, future research is recommended to further explore the safeguarding practices and functionality suggested to protect patients’ confidentiality while simultaneously supporting informal caregiving.

## Limitations

This study has several limitations. This study focuses on patients’ points of view and does not cover the medical perspective. Certain information might be essential from a medical point of view to record and share, despite the patient’s perspective. However, international experts, including HCPs, and patients have suggested establishing guidance for writing records on mental health notes.^72^ This is an ethical concern if individuals may not receive help because of the potential need to disclose sensitive topics to HCPs.

The study was restricted to one patient portal, My Kanta, in Finland, with a short three-week period and a low response rate of 0.37% (4719/1,262,708). Since some patient portal users did not logout from the patient portal, they may have not seen the survey invitation. In addition, the survey invitation was more difficult to recognize in mobile devices’ user interface, which may have affected lower respondent rate with younger users of patient portal. Since responding to the survey was voluntary, study respondents may represent users who were interested in the patient portal, able to use it, and currently active users of health care services. The response rate is similar with other national patient portal surveys such as previous studies in Finland (0.7%)^73^ and Sweden, where response rate was 0.61% with logged in users.^74^ In this study, the respondents were not representative of the population of Finland. In addition, the respondents were older than the average users of the patient portal. Moreover, the sample included more females, who are also more likely to use and access patient portals.^75,76^ The two patient groups we compared in this study, those who had received mental health care and those who had received other care. These groups were not similar in terms of sociodemographic factors, which meant that it was not possible to create subgroups without bias. The patterns of missing data did not seem to vary noticeable across the different groups.

To protect the respondents’ anonymity, identifying information was not collected. Researchers did not identify any potential duplicate submission during the data analysis. Respondents did not receive any compensation, and therefore, individual respondents had no reason to make multiple submissions to the survey.

Because the study was based on a relatively small sample, it was not possible to test statistical associations between the type of care and the qualitative responses. Furthermore, certain especially sensitive topics may not have been disclosed, as respondents could have considered them too sensitive to report. The survey did not include detailed information on whether respondents had a diagnosis or the type of diagnosis, which may have influenced responses when addressing especially sensitive topics.

Moreover, the study scope was limited to exploring what kinds of information are perceived as sensitive in PAEHRs; thus, findings on the potential consequences of sensitive information cannot be generalized to all patients, patient portal users or Finnish population. However, respondents spontaneously explained those potential negative consequences of sensitive information as part of their responses, without any prompting from the survey, which may highlight the importance of those potential negative consequences. Nonetheless, this study explores which mental health information was experienced as especially sensitive, even though it was not directly solicited from respondents. These results may also reflect how commonly patients experience certain sensitive information or health events in their lives when they might be especially vulnerable.

Sensitive information and the potentially stigmatizing nature of mental health information might depend on cultural differences,^77,78^ which were not further explored in this study. Additionally, the sensitive information may have positive, negative or neutral tone. Due to potential risk of respondents experiencing negative emotions or trauma when responding to the survey, questions were not mandatory. Moreover, certain information may be shared with other people or in another context, even though it would be experienced as sensitive.^21^ Future research is needed to explore which factors of sensitive health information in PAEHR may support care and which factors may lead to negative consequences.

### Implications for design

1) **Sensitive information should be handled and considered carefully when designing and implementing PAEHRs.** All health information is deemed sensitive. However, certain topics may be considered especially sensitive. Additionally, the context and concerns about privacy, such as who has access or how the information is used, may affect patients’ experiences. Thus, patients could have the possibility to limit access or mark certain information as especially sensitive within their PAEHR.
2) **Patients and caregivers should be actively involved in the design of PAEHRs.** To support the development of PAEHR, users could be heard during the developing process and use. The patient groups may have various needs and those may change depending on other factors such as changes in age or employment status. In addition, the sensitivity of certain topics may change on the basis of cultural context and patients’ individual conditions and health situation.
3) **HCPs could receive support in how to document sensitive information in the PAEHR.** Technical support for writing records such as word lists or phrases could help HCPs. Additionally, when tools are utilized to create notes, such as artificial intelligence (AI), it is recommended to approach potentially sensitive topics, such as mental health, with particular care.

## Conclusions

This explorative study offers valuable insight into the types of health information patients perceive as especially sensitive within their PAEHRs. Study participants noted that sensitivity often stems from the context of use, and potential consequences, such as the ability to access information, its impact on other aspects of care, or possible future harm in different contexts, including work life. In addition, patients described that certain health information could be stigmatizing. Among respondents, this concern was not limited to mental health related information but also extended to other types of health information.

All health information, particularly mental health-related information, could be handled and recorded especially carefully, as mental health information may be sensitive for patients despite the type of care. Additionally, patients who had received mental health care were more likely to perceive health information as especially sensitive. HCPs could handle health information with care to ensure that patients’ trust is not eroded, which may require improvements to current PAEHR systems. Moreover, sensitive information could be handled and considered carefully when designing and implementing PAEHRs. Patients could have the possibility to limit access or mark certain information as especially sensitive within their PAEHR. Additionally, certain topics could be marked automatically as sensitive within EHR systems.

HCPs could be involved when designing and implementing PAEHR. Professionals could receive support in how to document sensitive information in the EHR. Technical support for writing records, such as word lists or phrases, could help HCPs. Additionally, patients and caregivers could be actively involved in the design of PAEHRs. In addition, the sensitivity of certain topics may change on the basis of cultural context and patients’ individual conditions and health situation.

## Supplemental material

Supplemental material - What do patients consider sensitive health information? A cross-sectional survey of national patient portal users Supplemental material, Background Information Sensitive, for What do patients consider sensitive health information? A cross-sectional survey of national patient portal users by Saija Simola, Sari Kujala, Anna Kharko, Josefin Hagström, Charlotte Blease, Åsa Cajander, Rose-Mharie Åhlfeldt, Bo Wang, Bridget Kane, and Maria Hägglund in DIGITAL HEALTH.

Supplemental material - What do patients consider sensitive health information? A cross-sectional survey of national patient portal users Supplemental material, CHERRIES, for What do patients consider sensitive health information? A cross-sectional survey of national patient portal users by Saija Simola, Sari Kujala, Anna Kharko, Josefin Hagström, Charlotte Blease, Åsa Cajander, Rose-Mharie Åhlfeldt, Bo Wang, Bridget Kane, and Maria Hägglund in DIGITAL HEALTH.

Supplemental material - What do patients consider sensitive health information? A cross-sectional survey of national patient portal users Supplemental material, STROBE, for What do patients consider sensitive health information? A cross-sectional survey of national patient portal users by Saija Simola, Sari Kujala, Anna Kharko, Josefin Hagström, Charlotte Blease, Åsa Cajander, Rose-Mharie Åhlfeldt, Bo Wang, Bridget Kane, and Maria Hägglund in DIGITAL HEALTH.

Supplemental material - What do patients consider sensitive health information? A cross-sectional survey of national patient portal users Supplemental material, Survey Questions, for What do patients consider sensitive health information? A cross-sectional survey of national patient portal users by Saija Simola, Sari Kujala, Anna Kharko, Josefin Hagström, Charlotte Blease, Åsa Cajander, Rose-Mharie Åhlfeldt, Bo Wang, Bridget Kane, and Maria Hägglund in DIGITAL HEALTH.

## Data Availability

The quantitative datasets analyzed during the current study are available from the corresponding author upon reasonable request.*

## ORCID iDs

Saija Simola https://orcid.org/0000-0003-1819-1119

Sari Kujala https://orcid.org/0000-0001-5586-3725

Anna Kharko https://orcid.org/0000-0003-0908-6173

Josefin Hagström https://orcid.org/0000-0003-2835-0259

Charlotte Blease https://orcid.org/0000-0002-0205-1165

Åsa Cajander https://orcid.org/0000-0001-7472-2215

Rose-Mharie Åhlfeldt https://orcid.org/0000-0002-8607-948X

Bo Wang https://orcid.org/0000-0001-5667-8361

Bridget Kane https://orcid.org/0000-0003-3211-6529

Maria Hägglund https://orcid.org/0000-0002-6839-3651

## Appendix

### Abbreviations

AI: Artificial intelligence
EHR: Electronic health record
HCP: Health care professional
LGBTQIA+: Lesbian, Gay, Bisexual, Transgender, Queer, Intersex, and Asexual, +
PAEHR: Patient-accessible electronic health record
