# Supplementary material for: What do patients consider sensitive health information? A cross-sectional survey of national patient portal users
Source: Digit Health. 2026 Jun 9;12:20552076261459512. doi: 10.1177/20552076261459512 (PMC13250437; doi:10.1177/20552076261459512)
Supplement: Supplemental material - What do patients consider sensitive health information? A cross-sectional survey of national patient portal users [file sj-pdf-1-dhj-10.1177_20552076261459512.pdf]

Background information of the respondents and having reported especially sensitive information based on received type of care.

Sensitive, N (%)		Mental health care, N=692		Other care, N=3738		Total, N=4430	
		Yes	No	Yes	No	Yes	No
Age	15 – 17 years, N (%)	5 (50.0)	5 (50.0)	2 (33.3)	4 (66.7)	7 (43.8)	9 (56.3)
	18 – 19 years, N (%)	1 (33.3)	2 (66.7)	2 (40.0)	3 (60.0)	3 (37.5)	5 (62.5)
	20 – 24 years, N (%)	26 (76.5)	8 (23.5)	13 (56.5)	10 (43.5)	39 (68.4)	18 (31.6)
	25 – 34 years, N (%)	56 (58.3)	40 (41.7)	45 (40.5)	66 (59.5)	101 (48.8)	106 (51.2)
	35 – 44 years, N (%)	65 (54.6)	54 (45.4)	86 (40.0)	129 (60.0)	151 (45.2)	183 (54.8)
	45 – 54 years, N (%)	82 (58.2)	59 (41.8)	179 (42.6)	241 (57.4)	261 (46.5)	300 (53.5)
	55 – 64 years, N (%)	97 (53.3)	85 (46.7)	302 (33.0)	614 (67.0)	399 (36.3)	699 (63.7)
	65 – 74 years, N (%)	23 (31.5)	50 (68.5)	349 (24.6)	1067 (75.4)	372 (25.0)	1117 (75.0)
	75 – 84 years, N (%)	8 (32.0)	17 (68.0)	88 (15.6)	476 (84.4)	96 (16.3)	493 (83.7)
	85 years or more, N (%)	0 (0)	1 (100.0)	8 (17.4)	38 (82.6)	8 (17.0)	39 (83.0)
	No response, N (%)	5 (62.5)	3 (37.5)	4 (25.0)	12 (75.0)	9 (37.5)	15 (62.5)
Gender	Female	289 (53.5)	261 (47.5)	818 (30.4)	1870 (69.6)	1107 (34.2)	2131 (65.8)
	Male	64 (52.9)	57 (47.1)	231 (22.9)	779 (77.1)	295 (26.1)	836 (73.9)
	Other or no response, N (%)	15 (71.4)	6 (28.6)	29 (72.5)	11 (27.5)	44 (72.1)	17 (27.9)
Health care education	Yes	91 (54.8)	75 (45.2)	277 (34.2)	533 (65.8)	368 (37.7)	608 (62.3)
	No	269 (52.8)	240 (47.2)	776 (27.3)	2071 (72.7)	1045 (31.1)	2311 (68.9)
	No response, N (%)	8 (47.1)	9 (52.9)	25 (30.9)	56 (69.1)	33 (33.7)	65 (66.3)
Education	No formal education,	1 (33.3)	2 (66.7)	2 (16.7)	10 (83.3)	3 (20.0)	12 (80.0)
	Elementary school,	30 (50.8)	29 (49.2)	47 (12.2)	339 (87.8)	77 (17.3)	368 (82.7)
	12 years school - Upper secondary education,	110 (50.0)	110 (50.0)	242 (26.2)	682 (73.8)	352 (30.8)	792 (69.2)
	Higher vocational education (vocational diploma),	51 (46.8)	58 (53.2)	224 (25.8)	645 (74.2)	275 (28.1)	703 (71.9)
	Higher education ≤ 3 years (first cycle - bachelor),	88 (59.1)	61 (40.9)	224 (35.1)	415 (64.9)	312 (39.6)	476 (60.4)
	Higher education, >3 years (second cycle-master),	75 (62.5)	45 (37.5)	275 (28.9)	432 (61.1)	350 (42.3)	477 (57.7)

	Research, (third cycle) of higher education	4 (57.1)	3 (42.9)	28 (41.2)	40 (58.8)	32 (42.7)	43 (57.3)
	Something else	7 (41.2)	10 (58.8)	14 (18.2)	63 (81.8)	21 (22.3)	73 (77.7)
	No response	2 (25.0)	6 (75.0)	22 (39.3)	34 (60.7)	24 (37.5)	40 (62.5)
Employment	Full time	88 (53.7)	76 (46.3)	332 (37.1)	563 (62.9)	420 (39.7)	639 (60.3)
	Part time	33 (45.2)	40 (54.8)	69 (37.7)	114 (62.3)	102 (39.8)	154 (60.2)
	Student	39 (60.9)	25 (39.1)	37 (54.4)	31 (45.6)	76 (57.6)	56 (42.4)
	Retired	95 (47.5)	105 (52.5)	515 (22.7)	1754 (77.3)	610 (24.7)	1859 (75.3)
	Unemployed	39 (52.7)	35 (47.3)	48 (37.2)	81 (62.8)	87 (42.9)	116 (57.1)
	Not able to work	48 (60.0)	32 (40.0)	27 (36.0)	48 (64.0)	75 (48.4)	80 (51.6)
	Something else	25 (73.5)	9 (26.5)	43 (41.7)	60 (58.3)	68 (49.6)	69 (50.4)
	No response	1 (33.3)	2 (66.7)	7 (43.8)	9 (56.3)	8 (42.1)	11 (57.9)
Total	Received care	368 (53.2)	324 (46.8)	1078 (28.8)	2660 (71.2)	1446 (32.6)	2984 (67.4)

Totally 29 respondents did not report whether they have experienced health information that is especially sensitive.
