# Supplementary material for: What do patients consider sensitive health information? A cross-sectional survey of national patient portal users
Source: Digit Health. 2026 Jun 9;12:20552076261459512. doi: 10.1177/20552076261459512 (PMC13250437; doi:10.1177/20552076261459512)
Supplement: Supplemental material - What do patients consider sensitive health information? A cross-sectional survey of national patient portal users [file sj-pdf-2-dhj-10.1177_20552076261459512.pdf]

The survey methodology according to the CHERRIES checklist.

Item Category	Checklist Item	Description
Design		
	Survey design	The target group of the web-based survey was the national My Kanta patient portal’s logged-in patient users between January 24 and February 14, 2022.
Institutional Review Board		
	Approval	The study protocol was reviewed and approved by the Aalto University Research Ethics Committee (ethics approval number D/957/03.04/2020 Z).
	Informed consent	The survey invitation included study information and the privacy notice, and answering the questionnaire was regarded as informed consent to participate.
	Data protection	The survey was anonymous. The data is saved in a secured project folder at Aalto University.
Development and pre-testing		
	Development and testing	The survey was part of the NORDeHEALTH project in Finland, Estonia, Norway, and Sweden. Researchers from all 4 countries participated in the survey design. The survey was tested beforehand with 4 volunteer participants.
Recruitment process		
	Open vs closed survey	The survey invitation was sent only to the users who accessed their patient portal account between January 24 and February 14, 2022. Invitation to respond appeared to them at log-out.
	Advertising	The developer of the patient portal, the Social Insurance Institution of Finland, Kela, advertised the survey on Facebook with a short animated video. The video included texts: “Have you used the Omakanta service?” and “What is your experience of using Omakanta?”, and “Your opinion is important. Let’s make the digital health services better together”. In addition, instructions for responding were given.
Survey administration		

	Web/E-mail	The online survey link was provided in a pop-up window when the person logged out the patient portal.
	Context	Omakanta patient portal is provided and maintained by a government agency, the Social Insurance Institution of Finland (Kela).
	Mandatory/Voluntary	It was voluntary to fill in the survey.
	Incentives	No incentives were offered.
	Time	From January 24 to February 14, 2022.
	Adapted questioning	24 questions were visible only for the relevant group e.g. who has read their notes, has received mental health care, has received cancer care or has found errors or something offensive on their notes.
	Number of questions	45
	Number of pages	34
	Completeness check	None of the questions in the survey were compulsory.
	Review step	Respondents were able to us a back button.
Response rates		
	Unique site visitor	The questionnaire could be accessed only by logging out from your personal patient portal account. A unique visitor was not determined.
	Completion rate	83.10%
Preventing multiple entries		
	Cookies, IP, log file analysis	None were used.
	Registration	The survey invitation was sent only to the patient users who logged out from their personal patient portal account. Registration beyond that was not required, i.e. duplicate entries by respondents were not prevented.
Analysis		
	Handling of incomplete questionnaires	Only completed questionnaires were analyzed.
	Questionnaires submitted with an	Not measured.

	atypical timestamp	
	Statistical correlation	No methods were used to adjust the non-representative sample.
