# Supplementary material for: What do patients consider sensitive health information? A cross-sectional survey of national patient portal users
Source: Digit Health. 2026 Jun 9;12:20552076261459512. doi: 10.1177/20552076261459512 (PMC13250437; doi:10.1177/20552076261459512)
Supplement: Supplemental material - What do patients consider sensitive health information? A cross-sectional survey of national patient portal users [file sj-pdf-4-dhj-10.1177_20552076261459512.pdf]

Survey questions

How is your overall health?

Very good/ Good/ Fair/ Bad/ Very bad/ I don’t know / I don’t want to answer

In the last 2 years, have you received care from a doctor (GP or specialist) or other health professional for [select all that apply]

Mental health condition(s)/ Cancer/ Other health problem(s)/ No treatment

Do you consider some types of health information especially sensitive?

Yes/ No

If YES, **can you give an example of what type of health information that is most sensitive to you?**

(free text)

Gender

Female/ Male/ Other

Age

15 to 19 years old/ 20 to 24 years old/ 25 to 34 years old/ 35 to 44 years old/ 45 to 54 years old/ 55 to 64 years old/ 65 to 74 years old/ 75 to 84 years old/ 85 years old or older

Highest completed education

No formal education/ Elementary school/ 12 years school - Upper secondary education/ Higher vocational education/ Higher education ≤ 3 years/ Higher education, >3 years/ Doctoral education

Do you have a health care professional education?

Yes/ No

Which of the following best describes your current employment status? Choose the one that is most relevant.

Full time/ Part time/ Student/ Retired/ Unemployed/ Not able to work/ None of the above (free text)
